# Misregulation of PPAR Functioning and Its Pathogenic Consequences Associated with Nonalcoholic Fatty Liver Disease in Human Obesity

**DOI:** 10.1155/2012/107434

**Published:** 2012-12-09

**Authors:** Luis A. Videla, Paulina Pettinelli

**Affiliations:** ^1^Molecular and Clinical Pharmacology Program, Institute of Biomedical Sciences, Faculty of Medicine, University of Chile, Casilla 70000, Santiago 7, Chile; ^2^Ciencias de la Salud, Nutrición y Dietética, Facultad de Medicina, Pontificia Universidad Católica de Chile, 7820436 Santiago, Chile

## Abstract

Nonalcoholic fatty liver disease in human obesity is characterized by the multifactorial nature of the underlying pathogenic mechanisms, which include misregulation of PPARs signaling. Liver PPAR-**α** downregulation with parallel PPAR-**γ** and SREBP-1c up-regulation may trigger major metabolic disturbances between *de novo* lipogenesis and fatty acid oxidation favouring the former, in association with the onset of steatosis in obesity-induced oxidative stress and related long-chain polyunsaturated fatty acid n-3 (LCPUFA n-3) depletion, insulin resistance, hypoadiponectinemia, and endoplasmic reticulum stress. Considering that antisteatotic strategies targeting PPAR-**α** revealed that fibrates have poor effectiveness, thiazolidinediones have weight gain limitations, and dual PPAR-**α**/**γ** agonists have safety concerns, supplementation with LCPUFA n-3 appears as a promising alternative, which achieves both significant reduction in liver steatosis scores and a positive anti-inflammatory outcome. This latter aspect is of importance as PPAR-**α** downregulation associated with LCPUFA n-3 depletion may play a role in increasing the DNA binding capacity of proinflammatory factors, NF-**κ**B and AP-1, thus constituting one of the major mechanisms for the progression of steatosis to steatohepatitis.

## 1. Introduction

### 1.1. Epidemiologic Aspects

Nonalcoholic fatty liver disease (NAFLD) is considered the hepatic manifestation of metabolic syndrome (MetS) and has emerged as the most frequent cause of chronic liver disease worldwide, becoming the third most common indication for liver transplantation in order to rescue patients with end-stage liver disease [1, 2]. NAFLD encompasses a wide disease spectrum ranging from simple triacylglycerol (TAG) accumulation in hepatocytes (hepatic steatosis), which is defined by accumulation of liver fat (>5% per liver weight) in the presence of <20 g of daily alcohol consumption, to steatosis with inflammation (nonalcoholic steatohepatitis, NASH), fibrosis, and cirrhosis [2, 3]. Liver biopsy is the gold standard for diagnosis and has the additional benefit of distinguishing between NASH and simple steatosis, thus allowing for the staging of the degree of fibrosis [4]. NAFLD affects 17 to 33% in the general populations, whereas that of NASH affects 2% to 3% of the population [2, 5]. In obese subjects, NAFLD incidence reaches 60% to 90% and for NASH and hepatic cirrhosis 20% to 25% and 2% to 8%, respectively. In subjects with MetS, the prevalence of NAFLD is increased fourfold compared with those without the disease, and 30% of NAFLD subjects have MetS [6, 7]. In children population, an autopsy study found that 9.6% of the American population aged 2 to 19 years has NAFLD, and this figure increased to 38% among those who were obese [8]. 

Obesity is a state of chronic low-grade inflammation accompanied by excess fat storage deposited in tissues other than adipose tissue, including liver and skeletal muscle, which may lead to local insulin resistance (IR) and may stimulate inflammation, as in NASH [9]. Therefore, obesity and IR, both key features of the MetS, are intimately linked and strongly associated with NAFLD progression [3, 10]. 

### 1.2. Etiopathology of NAFLD

The primary metabolic abnormalities that lead to hepatic steatosis involve a lipotoxic response with an oxidative-stress component, nutritional factors, and alterations in the lipid metabolism of the liver, which result from the development of IR [3]. Hepatic fat accumulation, secondary to IR, develops when there is an imbalance in which fatty acid uptake and *de novo* synthesis exceed oxidation and secretion [11]. In this respect, the sources that contribute to fatty liver are (i) delivery of dietary fat to the liver (contribution to liver fat ~5%); (ii) delivery of extrahepatic nonesterified fatty acids (NEFAs) to the liver (contribution to liver fat ~60%); (iii) the remainder of liver fat accumulation is related to hepatic *de novo* lipogenesis, which is increased in obese patients [12]. 

The retention of FAs and TAGs within the hepatocytes that depends on IR and hyperinsulinemia leads to the production of free radicals at a mitochondrial level, capable of inducing lipid peroxidation, cytokine production, and hepatocyte necrosis [13], which may trigger NAFLD progression to the more severe state of NASH [2, 3]. 

The regulation of hepatic lipogenesis and FA oxidation is under rigorous control that involves a complex network of nuclear receptors, which regulate the expression of enzymes that participate in the lipid metabolism in a coordinated manner [11]. 

### 1.3. PPARs

The ligand-activated transcription factors belonging to the peroxisome proliferators-activated receptors (PPARs) are a subfamily of the steroid/thyroid/retinoid receptors superfamily. PPARs act as fatty acid sensors to control many metabolic programs that are essential for systematic energy homeostasis, including adipocyte differentiation, inflammation and energy homeostasis, lipoprotein metabolism, and FA oxidation, representing an important target for NAFLD [9, 14, 15]. The PPAR family consists of three members, namely, PPAR*α* (NR1C1 according to the unified nomenclature system for the nuclear receptor superfamily), PPAR*β*/*δ* (NR1C2), and PPAR*γ* (NR1C3), with two forms, *γ*1 and *γ*2, with differing amino termini, each encoded by different genes [14]. Similar to most members of the superfamily, all PPAR isoforms have a highly conserved structure. They are composed of five different domains, (i) an aminoterminal A/B domain involved in ligand-independent transactivation, which in other cases can regulate DNA binding, (ii) a two zinc-finger DNA-binding domain (DBD) responsible for half-site specificity of target gene recognition, (iii) a hinge region, (iv) a carboxy-terminal ligand-binding domain (LBD) with 60~70% homology between the subtypes, and (v) a transactivation domain, called AF2 (activation function 2) [16–18]. To control gene expression, PPARs heterodimerize with 9-cisRXR, which bind to peroxisome proliferator response elements (PPRE) located in the promoters of their targets genes. The canonical PPRE consists of two direct repeats AGGTCA separated by a single nucleotide so-called DR-1 element [14]. Activation of target gene transcription depends on the binding of the ligand to the receptor. Ligand binding induces a conformational change in the LBD of the receptor that facilitates recruitment of coactivator molecules. Unliganded nuclear receptors recruit corepressors N-CoR and SMRT. For PPAR:RXR heterodimer, binding of the ligand of either receptor can activate the complex, but binding of both ligands simultaneously is more potent [17, 19]. In this context, several studies have identified a series of endogenous and synthetic ligands for PPARs such as unsaturated fatty acids, oxidized low-density lipoproteins (LDL-ox), VLDL, metabolites derived from linoleic acid, fibrates, and thiazolidinediones [14, 20]. 

### 1.4. PPAR-***α***

Liver plays a pivotal role in lipid metabolism by upregulating the expression of numerous genes involved in FA uptake through membranes, FA activation, intracellular FA trafficking, FA oxidation, and ketogenesis, in addition to TAG storage and lipolysis. Furthermore, PPAR-*α* also governs the metabolism of glucose, lipoprotein, and amino acids besides inflammatory processes, mainly by downregulating gene expression via a transrepression mechanism [9, 21] (for a detailed review see [21]). PPAR-*α* is well expressed in metabolically active tissues including liver, heart, kidney, intestine, macrophages, and brown adipose tissue, and it has mostly been studied in the context of liver parenchymal cells, where it is highly expressed [21]. Although the functionality of PPAR-*α* was initially questioned due it lower expression compared with mouse liver [22], a recent study showed that in liver tissue and primary hepatocytes, PPAR-*α* expression levels in mice are similar to humans [23]. However, in this context, it has to be considered the presence of both a truncated splice variant of human PPAR-*α* that negatively interferes with wild-type PPAR-*α* activity [24] and polymorphic variants in the functional coding sequence of human PPAR-*α*, val227ala, and Leu162Val, which are implicated in NAFLD and IR but not with liver damage, respectively [25, 26]. Natural ligands of PPAR-*α* include a variety of FAs as well as numerous FA derivatives and compounds showing structural resemblance to FAs, including acyl-CoAs, oxidized FAs, eicosanoids, endocannabinoids, and phytanic acid [27–29]. Synthetic PPAR-*α* ligands include fibrates such as gemfibrozil, bezafibrate, clofibrate, fenofibrate, and Wy14643, drugs that are used in the treatment of dyslipidemia primarily associated with type 2 diabetes mellitus [21]. 

### 1.5. PPAR-***γ***

PPAR-*γ* is the master regulator in the control of genes involved in lipogenic pathways of adipocytes, promoting the uptake of FAs and the differentiation of the adipocyte, with the consequent increase in the cellular content of TAGs and reduction in the FA delivery to the liver [17]. Target genes of PPAR-*γ* are involved in adipocyte differentiation, lipid storage, and glucose metabolism and include lipoprotein lipase, CD36, adipocyte FA binding protein aP2, FA transport protein, acyl-coA synthetase, phosphoenolpyruvate carboxykinase, aquaporin 7, and citrate carrier [9, 30, 31]. PPAR-*γ* also confers sensitization to insulin through the transcriptional activation of the adiponectin gene in adipocytes, up-regulating its expression [32]. Ligands for PPAR-*γ* include specific polyunsaturated fatty acid (PUFA) metabolites, several eicosanoids, and synthetic compounds with very high (nanomolar) affinity such as thiazolidinediones [17, 29]. 

Increased PPAR-*γ* expression is a feature of the steatotic liver and several studies attribute a causal role of PPAR-*γ* in steatosis development by mechanisms involving activation of lipogenic genes and *de novo* lipogenesis [33]. In humans, PPAR-*γ* is much more abundant in adipose cells; yet reasonable levels of PPAR-*γ* mRNA can also be found in other organs including skeletal muscle, colon, lung, and placenta. In contrast to adipose tissue, liver and heart express very little PPAR-*γ*; however, under certain pathological conditions, these tissues can express considerable amounts of PPAR-*γ* that have significant impacts on metabolic homeostasis and tissue function [34]. 

Studies addressing the expression of PPAR-*γ* in obese subjects revealed an increased adipose tissue expression of the splice variants PPAR-*γ*1 and PPAR-*γ*2, compared with lean subjects, suggesting that under pathological conditions and different nutritional situations, regulation of the human PPAR-*γ* expression may change [35]. In some infectious diseases such as hepatitis B and C viruses, multiple observations suggest that liver steatosis is a common histological characteristic, in which an increase in the expression and/or activity of PPAR-*γ* could contribute to the regulation of lipid synthesis [36–38]. Furthermore, similar to PPAR-*α*, it has to be considered PPAR-*γ* variants, considering that Pro12Ala and C1431T polymorphisms alter the susceptibility to hepatic steatosis, lobular inflammation, and fibrosis in humans with NAFLD. It was suggested that subjects with a haplotype containing both minor Pro12Ala and C1431T alleles are at reduced risk for NAFLD, and its histological features are associated with NASH [39]. Similar results have been found in Chinese population [40], which is in agreement with previous results associating Pro12Ala variant with increased insulin sensitivity, lower body mass and protection from type 2 diabetes [41–43].

### 1.6. PPAR-***δ***

Due to its ubiquitous expression profile, much less is known about PPAR-*δ* compared to PPAR-*α* and PPAR-*γ* in relation to human obesity and NAFLD. Studies from a decade ago showed that insulin-resistant obese rhesus monkeys normalized fasting glucose and insulin, increased high-density lipoprotein-cholesterol, and reduced low-density lipoprotein cholesterol after treatment with the potent and specific PPAR-*δ* agonist GW501516, which is approximately 1200 times more selective for PPAR-*δ* than the *α* and *γ* receptors [44]. Studies in an animal model of adenovirus-mediated hepatic PPAR-*δ* overexpression showed that PPAR-*δ* regulates lipogenesis and glucose utilization for glycogen synthesis. These effects could result in hepatic protection from free FA-mediated damage, possibly due to the generation of protective mono-unsaturated FA and lowering lipotoxic saturated FA levels [45]. Overweight and obese men subjected to PPAR-*δ* agonists GW501516 or MBX-8025 improved insulin sensitivity and decreased fasting plasma TAGs, NEFAs, apoB-100, and LDL-cholesterol concentrations, with diminished liver fat content quantified by magnetic resonance imaging (MRI) [46–49]. Furthermore, recent studies showed that enhanced inflammation in visceral adipose tissue (VAT) is accompanied by a reduction in SIRT1 protein levels and PPAR-*δ* activity, in association with NF-*κ*B activation, in morbidly obese IR patients compared with normal and overweight subjects, suggesting interplay between PPAR-*δ* and NF-*κ*B [50]. However, this contention and the mechanisms underlying PPAR-*δ* effects remain to be studied in the liver of obese patients.

 Collectively, discussed data point to various molecular mechanisms underlying NAFLD, some of which are modulated by PPARs. The aim of this work is to review the alterations of PPAR functioning and its pathogenic consequences associated with NAFLD in human obesity. 

## 2. The Role of PPAR-***α*** Downregulation in Liver Steatosis

**Figure 1:**
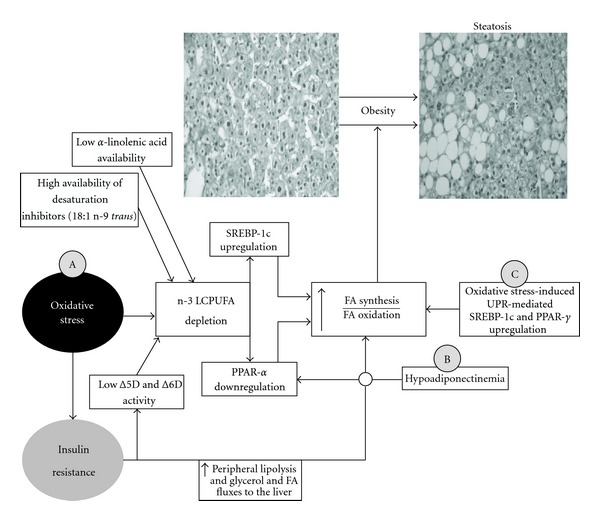
Obesity-induced liver oxidative stress (A), hypoadiponectinemia (B), and endoplasmic reticulum stress (C) as factors leading to hepatic steatosis in nonalcoholic fatty liver disease. *Abbreviations: *Δ5(6)D: delta-5(6) desaturase; FA, fatty acid; LCPUFA: long-chain polyunsaturated fatty acid; PPAR-*α*(*γ*): peroxisome proliferator-activated receptor-*α*(*γ*); SREBP-1c: sterol regulatory element binding protein-1c; UPR: unfolded protein response.

Simple TAG accumulation in hepatocytes or steatosis is an early hallmark in NAFLD associated with obesity that is characterized by the multifactorial nature of the underlying pathogenic mechanisms, including the development of oxidative stress and insulin resistance [3, 52, 53], which provides the setting for further hepatic injury [54]. In this respect, the concept of nutritional or dietary oxidative stress has been introduced to denote an imbalance between the prooxidant load and the antioxidant defence, resulting from excess oxidative load or inadequate supply of the organism with nutrients [55]. Prolonged consumption of calorie-enriched diets stimulates fatty acid (FA) synthesis from glucose, and FAs in excess are converted into TAGs and store as lipid droplets within hepatocytes (Figure 1) [56]. FA overloading in the liver may favour high rates of FA oxidation due to substrate pressure, with consequent reactive oxygen species (ROS) generation [3]. This contention is supported by studies in J774.2 macrophages, which upon TAG overload generate ROS at mitochondrial complex I of the respiratory chain, coupled to higher FA *β*-oxidation, with concomitant induction of cellular necrosis, features that are diminished by antioxidants [13]. In agreement with these views, the liver of obese NAFLD patients with steatosis exhibits major changes in oxidative stress-related parameters. These include (i) a diminished antioxidant potential (glutathione (GSH) depletion and reduced superoxide dismutase (SOD) activity) [57]; (ii) an increased free-radical activity (higher lipid peroxidation) [57–59], protein oxidation [57], and 3-nitrotyrosine reactivity [60]; (iii) Kupffer-cell activation (increased lipid peroxidation potential and superoxide radical (O_2_^•−^) generation, implying NADPH oxidase (NOX2) activation) [61]; (iv) a consequent reduction in the systemic antioxidant capacity of plasma [57] with higher lipid peroxidation indicators [62], thus evidencing the onset of oxidative stress (Figure 1A). Overnutrition-induced ROS generation might represent a triggering mechanism for the onset of insulin resistance [63, 64], in addition to the accumulation of lipids such as free FAs (FFAs) [65, 66]. This proposal points to the activation of several stress-sensitive serine/threonine kinases by ROS and FFAs, which upon phosphorylation of the insulin receptor and/or the insulin receptor substrate proteins, achieve derangement of insulin-stimulated tyrosine phosphorylation resulting in insulin resistance [63–66]. 

**Figure 2:**
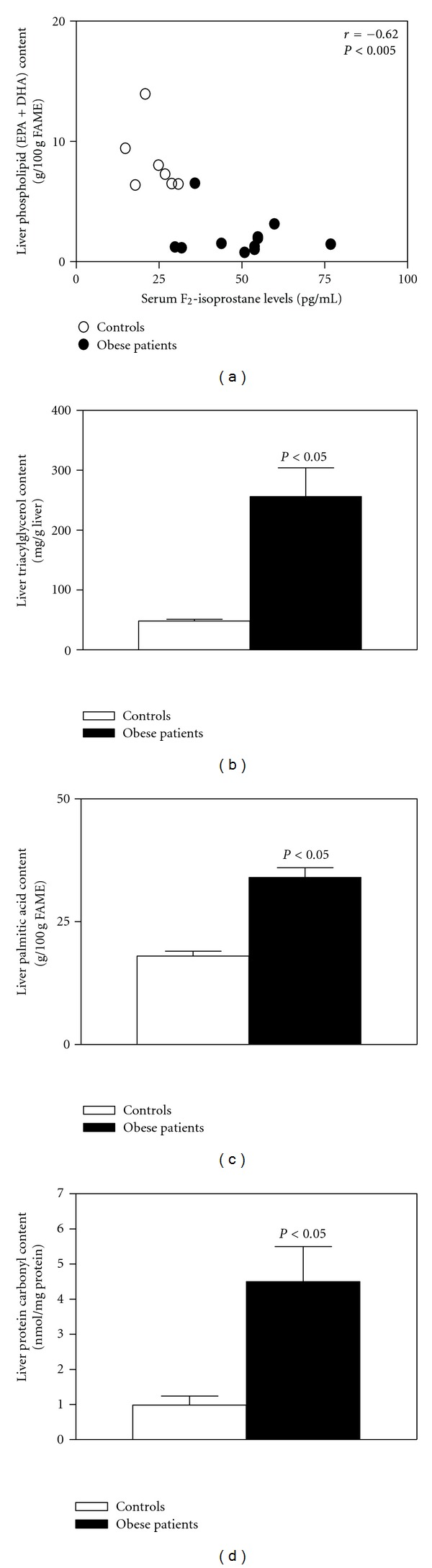
Correlation between liver phospholipid content of LCPUFA n-3 and F_2_-isoprostane levels in serum as index of oxidative stress (a) and contents of liver triacylglycerols (b), palmitic acid (c), and protein carbonyls (d) in control subjects and obese patients with steatosis. LCPUFA n-3 content corresponds to eicosapentaenoic acid (EPA) plus docosahexaenoic acid (DHA), expressed as g/100 g fatty acid methyl esters (FAME). Correlation in (a) was carried out by Spearman rank order correlation coefficient (unpublished data). Data (means ± SEM; 10 controls and 8 obese patients with steatosis) presented in (b), (c), and (d) were adapted from Araya et al., 2004 [51].

 Development of cellular oxidative stress leads to the production of oxidized products of biomolecules such as DNA bases, aminoacid residues in proteins, and PUFAs in membrane phospholipids [67]. In the latter case, long-chain PUFAs (LCPUFAs) of the n-3 series, namely, eicosapentaenoic acid (EPA, 20:5n-3) and docosahexaenoic acid (DHA, 22:6n-3), are the most susceptible to free-radical attack, considering that their respective rate constants for lipid peroxidation initiation are about 7- to 10-fold higher than that for linoleic acid (LA, 18:2n-6) taken as unity [68]. Assessment of the FA pattern of the liver of obese NAFLD patients revealed a significant depletion of LCPUFA n-3 (EPA plus DHA) levels [51, 69, 70]), a parameter that correlates with the levels of LCPUFA n-3 in erythrocytes [69] and that significantly recovers after weight loss [71]. Liver LCPUFA n-3 depletion in obesity may be related to higher utilization due the prevailing high oxidative stress status [57, 72] (Figure 1A), a contention that is supported by the significant inverse correlation established between liver phospholipid LCPUFA n-3 content and serum F_2_-isoprostane levels, as index of free-radical activity (Figure 2(a)). Under these conditions, the nonenzymatic oxidative decomposition of LCPUFA n-3 to J_3_-isoprostane derivatives [73] can occur; however, utilization of LCPUFA n-3 by cyclooxygenase-2/5-lipoxygenase pathway and/or the cytochrome P450 NADPH-dependent epoxygenase system [74] cannot be discarded. In addition to enhanced liver LCPUFA n-3 utilization, depletion of LCPUFA n-3 in NAFLD is associated with defective hepatic capacity for desaturation of the LCPUFA n-3 essential precursor *α*-linolenic acid (*α*-LA, 18:3n-3). (i) Livers from NAFLD patients show a significant diminution in the hepatic activity of Δ-5 and Δ-6 desaturases (Δ-5D and Δ-6D) [75] and in the (20 : 5 + 22 : 6)n-3/18:3n-3 product/precursor ratio [51]. These parameters exhibit inversed correlations with the HOMA index [75], pointing to coordinate downregulation of Δ-5D and Δ-6D expression by insulin resistance (Figure 1A) [76, 77]. (ii) Dietary imbalance, as determined by the abdominal adipose tissue PUFA levels as biomarker of dietary intake [78], involves decreased consumption of *α*-LA and higher-than-normal intake of *trans* FAs (elaidic acid, 18:1n-9 *trans*), effective Δ-6D inhibitors (Figure 1A) [51].

 Under physiological conditions, LCPUFAs n-3 and/or their oxidized metabolites regulate hepatic lipid metabolism acting as (i) ligands of PPAR-*α* promoting the expression of genes encoding for proteins involved in FA oxidation at mitochondrial, peroxisomal, and microsomal levels, FA binding in cells, and lipoprotein assembly and transport [20] and (ii) downregulators of the lipogenic transcription factor SREBP-1c expression and activation [79–81]. Therefore, LCPUFA n-3 depletion in the liver of obese NAFLD patients might favour FA and TAG synthesis over FA oxidation, promoting hepatic steatosis (Figure 1A), with major changes in the mRNA expression of transcription factors controlling liver lipid metabolism. The latter view is evidenced by the increased mRNA expression of SREBP-1c inducing lipogenic genes such as fatty acid synthase (FAS), the concomitant reduction in that of PPAR-*α* controlling FA oxidation (carnitine palmitoyltransferase-1a; CPT-1a), with the consequent enhancement in the hepatic SREBP-1c/PPAR-*α* ratios denoting a prolipogenic status [70]. This condition may also involve diminution in TAG export from the liver via very-low density lipoprotein (VLDL) due to decreased production of apolipoprotein B-100 [82], which is upregulated by LCPUFA n-3 and PPAR-*α* activation [83, 84]. The above contention is further strengthened by the substantial enhancement in the LCPUFA n-6/n-3 ratio observed in liver phospholipids [73, 85], considering that LCPUFA n-3 are more effective PPAR-*α* activators than LCPUFA n-6 [79]. In agreement with these findings obtained in the liver, of obese patients, nutritional disequilibrium at the expense of PUFA n-3 in mice subjected to a PUFA n-3 depleted diet-induced hepatic SREBP-1c and lipogenesis up-regulation, with significant depression of FA oxidation and steatosis development [86]. 

 In addition to the prosteatotic mechanism underlying NAFLD with development of oxidative stress and LCPUFA n-3 depletion triggering liver SREBP-1c upregulation and PPAR-*α* downregulation (Figure 1A), alterations in the signaling pathway of adiponectin may also play a role [70, 87] (Figure 1B). Adiponectin, an adipokine secreted by adipocytes in reverse proportion to the body mass index [88], exerts beneficial effects through actions on several tissues, leading to reduction of body fat, improvement of hepatic and peripheral insulin sensitivity, and increased FA oxidation [32, 89]. In the liver, adiponectin binds to the integral membrane proteins AdipoR1 and AdipoR2 acting as receptors for the globular and full-length forms of the adipokine [89]. Although the signaling pathway triggered by adiponectin is not completely understood, current views suggest that most of its cellular effects are mediated by the activation of AMP-activated protein kinase (AMPK) [90]. This is achieved by APPL1 (adaptor protein containing phosphotyrosine binding, pleckstrin homology domains, and leucine zipper 1) that couples adiponectin receptors to AMPK activation [91], with the sequential activation of p38 mitogen-activated protein kinase (p38 MAPK) [92] that phosphorylates PPAR-*α*, thus increasing its association with PPAR-*α* coactivator-1*α* and the transcriptional activity of PPAR-*α* [93]. Consequently, the expression of PPAR-*α* target genes encoding for acyl-CoA oxidase, CPT-1a, and fatty acid binding protein 3 is upregulated [91]. Therefore, diminution in the circulating levels of adiponectin [79, 87, 94] and in the hepatic expression of adiponectin and AdipoR2 [95] observed in obese NAFLD patients might contribute to liver PPAR-*α* downregulation (Figure 1B), representing an alternate reinforcing prolipogenic mechanism in addition to that related to LCPUFA n-3 depletion (Figure 1A). 

## 3. Liver PPAR-***γ*** Upregulation as a Steatotic Signaling Mechanism

The specific PPAR subtype PPAR-*γ* is mainly expressed in the white and brown adipose tissue [96], where it controls the expression of genes related to lipogenesis, promoting cell differentiation, FA uptake, and TAG accumulation, which reduces FA delivery to the liver [97]. In the human liver, PPAR-*γ* is expressed at a level that is 9–12% of that of adipose tissue [35]; however, enhanced expression levels are associated with induction of PPAR-*γ*-responsive genes related to lipid metabolism [98]. These include (i) lipoprotein lipase, (ii) proteins involved in FA uptake and intracellular binding and transport, such as FA translocase (FAT/CD36), FA binding proteins 1, 4, and 5 (FABP1, FABP4, and FABP5), and FA transport proteins 2 and 5 (FATP2 and FATP5), and (iii) liver X receptor, which favours both PPAR-*γ* and FAT/CD36 expression [14, 99]. Studies in the liver of obese NAFLD patients revealed significant upregulation of PPAR-*γ* mRNA levels over those in lean control subjects [94], in agreement with data assessing the PPAR-*γ*2 isoform [100]. Furthermore, liver PPAR-*γ* upregulation coincided with that of SREBP-1c, parameters that showed a significant direct correlation and that constitute a reinforcing lipogenic mechanism [94, 101]. This contention is supported by the differential lipogenic gene expression pattern exhibited by both transcription factors. Under condition of insulin resistance, higher mobilization of nonesterified FAs from peripheral tissues to the liver occurs [102, 103], which may be efficiently taken up and subjected to intracellular trafficking for metabolic processing, due to PPAR-*γ*-dependent upregulation of liver FAT/CD36 and FATP5, respectively [102]. Thus, enhancement in *de novo* TAG biosynthesis can be achieved [12, 104], which may be contributed by *de novo* FA biosynthesis due to SREBP-1c-dependent induction of acetyl-CoA carboxylase, FAS, and stearoyl-CoA desaturase-1 observed [94, 105]. 

 Upregulation of liver PPAR-*γ* can be achieved by a ligand-dependent process including LCPUFA n-3 binding [106]; however, this mechanism does not seem to play a role in obesity-induced PPAR-*γ* activation due to the substantial depletion of LCPUFA n-3 reported [51, 69, 70]. Although development of insulin resistance is likely to involve loss of the regulatory actions of insulin on hepatocellular carbohydrate, protein, and lipid anabolism, FA and TAG biosynthesis is preserved [53, 107]. It is therefore likely that other mechanisms may play a role in the prolipogenic status observed in obese, insulin-resistant, hyperinsulinemic individuals involving PPAR-*γ* and SREBP-1c [70, 92]; the endoplasmic reticulum (ER) stress is one of them [108]. The ER is the cellular compartment for protein synthesis, folding, assembly, and trafficking, as well as for TAG, phospholipid, and sterol biosynthesis [108, 109]. Under several stress conditions, accumulation of abnormally folded proteins triggers the unfolded protein response (UPR), to relieve the ER from the accumulation of misfolded proteins and avoid loss of protein function [108, 109]. A short-lasting UPR reestablishes folding capacity; however, under prolonged or sustained conditions, ER stress changes from cellular survival promotion to liver injury development [108, 110]. The UPR is mediated by three ER transmembrane proteins, namely, (i) double-stranded RNA-activated protein kinase (PKR-) like endoplasmic reticulum kinase (PERK); (ii) inositol requiring enzyme 1 (IRE1); (iii) activating transcription factor 6 (ATF6) [108, 111, 112]. These UPR transducers are normally inhibited by the ER chaperone BiP/Grp78 (binding immunoglobulin protein/glucose regulated protein 78) [113], which upon accumulation of misfolded proteins in the ER lumen dissociates from the luminal domains of PERK, IRE1, and ATF6 allowing their activation [108]. UPR is induced by several stress conditions, including reduced capacity for protein glycosylation or disulfide bond formation, nutrient deprivation, viral infections, and increased FA availability or ROS generation, which led to abnormal protein folding [108, 114]. As already discussed in Section 2, human obesity is characterized by TAG (Figure 2(b)) and saturated FA (palmitic acid; Figure 2(c)) overload in the liver, determining high rates of FA oxidation and ROS generation [3], which is associated with 4-fold increase in hepatic protein carbonylation (Figure 2(d)), as a measure of protein oxidation by ROS [115]. Protein damage by ROS is complex, irreversible and involves various oxidative modifications of amino acid residues in proteins, which may lead to protein unfolding and rapid degradation [115–117]. Thus, under conditions of hepatic palmitate overload (Figure 2(c)) and ROS-dependent protein carbonylation (Figure 2(d)), ER stress is likely to be induced in the liver of obese NAFLD patients. This contention is in agreement with the elevated hepatic levels of BiP/Grp78 and of phosphorylated eukaryotic translation-initiation factor 2*α* (eIF2*α*) as components of the PERK signaling pathway [118, 119], which are significantly diminished after weight loss [118]. In addition to the liver, adipose tissue from obese patients also exhibits increased parameters related to ER stress, evidencing the activation of the PERK [118, 120], IRE1 [111, 118], and TAF6 [120] signaling pathways. These findings suggest the involvement of the UPR in lipogenesis leading to hepatic steatosis (Figure 1C), in addition to obesity-induced oxidative stress-related LCPUFA n-3 depletion, insulin resistance (Figure 1A), and hypoadiponectinemia (Figure 1B). Interestingly, ER stress has been associated with ROS generation [121] that may contribute to the oxidative stress status developed in the liver of obese patients [3, 57, 72]. The proposed mechanisms involving ER stress induced (i) sustained Ca^2+^ release from the ER and mitochondrial Ca^2+^ accumulation with promotion of ROS production [114, 121], and (ii) oxidative folding of nascent proteins by protein disulfide isomerase (PDI) coupled to ER-oxidoreductin 1 (Ero1) operation [114, 121, 122]. However, several important mechanistic questions remain to be addressed regarding the role of UPR in obesity-related liver disease [114] and oxidative stress development [121]. 

## 4. Liver PPAR-***α*** Downregulation: Proinflammatory Connotations

**Figure 3:**
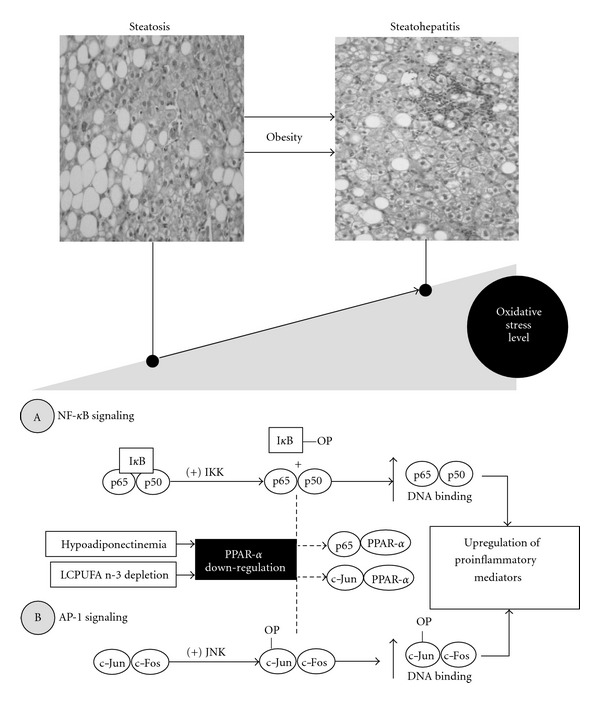
Interrelationships between the level of oxidative stress and PPAR-*α* downregulation in the progression of steatosis to steatohepatitis associated with obesity involving NF-*κ*B (A) and AP-1 (B) signaling. *Abbreviations:* AP-1: activating protein-1 (c-Jun-cFos; c-Jun-OP, phosphorylated c-Jun); I*κ*B: inhibitor of *κ*B (I*κ*B-OP, phosphorylated I*κ*B); IKK: I*κ*B kinase; JNK: c-Jun N-terminal kinase; LCPUFA, long-chain polyunsaturated fatty acid; NF-*κ*B: nuclear factor-*κ*B (p65-p50); PPAR-*α*: peroxisome proliferator-activated receptor-*α*. Solid arrows indicate enhanced contribution, whereas broken arrows indicate reduced contribution.

Liver oxidative stress status, a major mechanism associated with the pathogenesis of steatosis (Figure 1), is exacerbated in obese patients with steatohepatitis (Figure 3). This is evidenced by (i) diminution of hepatic catalase activity, in addition to SOD reduction and GSH depletion already observed in steatosis [57]; (ii) upregulation of the cytochrome P450 2E1 isoform (CYP2E1) and higher *in vivo* chlorzoxazone hydroxylation catalyzed by CYP2E1, changes that are not observed in steatosis [123]; (iii) further increases in liver nitrotyrosine immunoreactivity [60], hepatic 4-hydroxynonenal (marker of lipid peroxidation) and 8-hydroxydeoxyguanosine (marker of oxidative DNA damage) immunostaining [124], Kupffer-cell-dependent O_2_^•−^ generation, and lipid peroxidation extent [61] (Figure 3). These changes observed in the liver of steatohepatitis subjects are paralleled by a further decrease in the antioxidant capacity of plasma over that in steatosis [57], which correlates with higher systemic levels of lipid peroxidation products [62, 125–127]. Liver oxidative stress in steatohepatitis is related to several contributory factors, including upregulation of the highly prooxidant CYP2E1 [58, 123, 128], hepatic mitochondrial dysfunction [129, 130], and mixed inflammatory-cell infiltration and Kupffer-cell activation, involving upregulation of NOX2 [61]. The high prooxidant status attained in steatohepatitis was observed concomitantly with significant enhancement in the DNA binding capacity of hepatic nuclear factor-*κ*B (NF-*κ*B) [131, 132] and activating protein 1 (AP-1) [131], redox-sensitive transcription factors that upregulate the expression of proinflammatory mediators at the Kupffer-cell level (Figure 3) [3]. These parameters were not modified in patients with simple steatosis, in relation to controls [131]. 

ROS activate NF-*κ*B through the classical or canonical inhibitor of *κ*B (I*κ*B) kinase (IKK) complex pathway, which depends on NF-*κ*B essential modulator (NEMO) or IKK*γ*, IKK*β* activation, nuclear localization of p65-p50 dimers and is associated with inflammation (Figure 3A) [133, 134]. In addition, AP-1 signaling requires *de novo* synthesis of c-Jun and c-Fos proteins, followed by phosphorylation of the c-Jun component by activated c-Jun N-terminal kinase (JNK) (Figure 3B), which requires ROS-mediated inhibition of JNK-inactivating phosphatases [135]. At the nuclear level, both NF-*κ*B and AP-1 may form heterodimers with PPAR-*α*, leading to the formation of the transcriptionally inactive complexes p65-PPAR-*α* and c-Jun-PPAR-*α* [53]. Thus, obesity-induced diminution in both liver PPAR-*α* expression and PPAR-*α* activation related to LCPUFA n-3 depletion may be considered as a proinflammatory mechanism [70], due to the reduced antagonizing action of PPAR-*α* downregulation on NF-*κ*B and AP-1 activation. This contention is supported by the significant inverse correlations established for liver NF-*κ*B and AP-1 DNA binding with PPAR-*α* mRNA levels observed in control subjects and obese NAFLD patients with steatohepatitis [136]. Furthermore, significant 7.8-fold and 15.1-fold enhancements in the hepatic NF-*κ*B/PPAR-*α* and AP-1/PPAR-*α* ratios are observed in steatohepatitis over control values, respectively, pointing to a major disturbance in signaling pathways triggering a proinflammatory status in the liver of obese patients (Figure 3) [136]. The latter state may be reinforced by three additional mechanisms, namely, (i) TNF-*α* up-regulation [61, 137–139] in response to the initial NF-*κ*B activation, which signals through the TNF-*α* receptor 1 and the canonical pathway and/or by TNF-*α*-induced ROS production at the mitochondrial level that activates JNK, enhancing AP-1 DNA binding capacity [133, 134]; (ii) development of endotoxemia [140], with increasing plasma levels of lipopolysaccharide (LPS) triggering toll-like receptor 4 (TLR4) [141], recruitment of several adaptor molecules, and activation of transforming growth factor *β*-activated kinase 1 (TAK1) leading to IKK and JNK phosphorylation and NF-*κ*B and AP-1 activation [141, 142]; (iii) induction of the ER stress, with upregulation of both the PERK/eIF2*α* pathway [118, 119] achieving NF-*κ*B activation [143] and the IRE1 pathway leading to JNK/AP-1 activation [111, 118, 119]. Although ER stress can activate NF-*κ*B and JNK/AP-1, activation by other mechanisms is also possible, and further studies are needed to establish their relative importance in the development of steatohepatitis in human obesity. 

## 5. Conclusions

Prolonged liver oxidative stress in human obesity is associated with development of a wide disease spectrum ranging from steatosis to steatosis with inflammation, fibrosis, and cirrhosis (nonalcoholic steatohepatitis), a redox imbalance showing a functional interdependency with insulin resistance [3]. Disease mechanisms might involve (i) the initial ROS production due to lipotoxicity with the onset of steatosis (Figure 1); (ii) exacerbation of ROS generation due to concurrence of mitochondrial dysfunction, microsomal CYP2E1 induction, and inflammatory-cell activation (Figure 3). Misregulation of inflammatory cytokine, adipokine, and chemokine signaling may reinforce the initial mechanisms of ROS production and insulin resistance, representing determinant factors in the progression of steatosis to steatohepatitis. In this setting, alterations in the expression and/or activation in hepatic PPARs may play crucial pathogenic roles, considering their importance in energy homeostasis and inflammation [106]. 

Liver PPAR-*α* downregulation and substantial enhancement in the hepatic SREBP-1c/PPAR-*α* mRNA content ratio represent major metabolic disturbances between *de novo *lipogenesis and FA oxidation favouring the former, as a central issue triggering liver steatosis in obesity-induced oxidative stress and insulin resistance. The prosteatotic action of PPAR-*α* downregulation may be reinforced by PPAR-*γ* upregulation favouring hepatic FA uptake, binding, and transport, representing a complementary lipogenic mechanism to SREBP-1c induction leading to *de novo* FA synthesis and TAG accumulation. In addition, PPAR-*α* downregulation may play a significant role in enhancing the DNA binding capacity of proinflammatory factors NF-*κ*B and AP-1 in the liver of obese patients, thus constituting one of the major mechanisms for the progression of simple steatosis to steatohepatitis. In the past, PPARs have been studied as drug targets for the management of NAFLD in obesity and the broader MetS [53]. However, PPAR-*α* agonists such as fibrates used to diminish steatosis and inflammatory scores in human steatohepatitis revealed poor effectiveness, thiazolidinediones have weight gain limitations, whereas that of dual PPAR-*α⁄γ* agonists has safety concerns [53]. Considering the negative correlation established between liver SREBP-1c/PPAR-*α* ratios and LCPUFA n-3 levels in control and obese subjects [70], which points to liver LCPUFA n-3 depletion as a major factor directing FAs toward TAG storage, LCPUFA n-3 supplementation was used as PPAR-*α* targeting. Supplementation with either fish oil, seal oil, or purified LCPUFA n-3 diminished steatosis scores, as evidenced by ultrasonography [144–147] or by determination of lipid content in posttreatment liver biopsies [148]. Furthermore, improvement in liver function markers [144–148], TAGs [145, 146] and TNF-*α* [145] levels in serum were observed after LCPUFA n-3 administration. These data were recently included in a larger meta-analysis comprising nine studies involving 355 individuals, which concluded that LCPUFA n-3 supplementation in human NAFLD patients is associated with a positive effect on liver fat [149]. In addition, positive anti-inflammatory outcome is also observed [144–148], which may include (i) PPAR-*α* activation and further inhibitory action on NF-*κ*B and AP-1 signaling [150]; (ii) EPA and DHA metabolism by the cyclooxygenase-2/5-lipoxygenase pathway to produce E(D) resolvins and protectin D1 as anti-inflammatory mediators [151]; (iii) EPA and DHA oxygenation by cytochrome P450 NADPH-dependent epoxygenases, with production of epoxy derivatives with potent anti-inflammatory effects [151]. LCPUFA n-3 effects on liver inflammation and fibrosis are being currently addressed by several clinical trials [152].
